# A case of hypotension after intranasal adrenaline infiltration causing a clinical dilemma during the intraoperative period

**DOI:** 10.4103/0019-5049.76595

**Published:** 2011

**Authors:** Shyam Bhandari, Mozammil Shaffi, S Bano, Suhail Sarwar Siddiqui, Jahangir Ahmad

**Affiliations:** Department of Anaeasthesiology, J.N. Medical College, Aligarh Muslim University, Aligarh, India

**Keywords:** Adrenaline, anaphylaxis, Bezold Jarisch reflex, hypotension, β2 receptor

## Abstract

Solutions containing adrenaline are widely used for presurgical infiltration. Haemodynamic changes associated with its use are well documented in the literature. Prolonged intraoperative hypotension after subcutaneous infiltration of diluted adrenaline is an uncommon scenario. We believe that our case of the prolonged episode of hypotension was secondary to infiltration of nasal septum with a high concentration of adrenaline. As β2 receptor activation leads to skeletal muscle vasodilation, a decrease in preload may have lead to profound hypotension. Postoperatively, the patient was examined and any autonomic or endocrinological pathology was ruled out.

## INTRODUCTION

The routine use of different dilutions of adrenaline with or without local anaesthetics is a universally accepted technique in ENT surgeries. Some studies[1,2] have concluded that there is no significant haemodynamic changes while others have demonstrated significant transient changes in heart rate and mean arterial pressure.[3–6]

Pulse rate (PR) and mean arterial pressure (MAP) may increase or decrease depending on the plasma concentration of adrenaline attained and activation of specific receptors sites.[7,8] β2, β1 and *α*1 receptors are activated at 1-2 *μ*g/min i.v., 4 *μ*g/min i.v. and 10-20 *μ*g/min i.v., respectively. Recently, we experienced a unique and rare complication of adrenaline infiltration in which prolonged hypotension was noticed after subcutaneous adrenaline infiltration.

## CASE REPORT

A young, 25-year-old, 50 kg in weight, male was posted for surgical correction of deviated nasal septum under general anaesthesia. Preoperative vitals were stable (pulse rate 64/min, regular and blood pressure (BP) 110/76 mmHg). Intravenous line was secured with 20 G cannula and lactated ringer was started. Monitoring was done with noninvasive blood pressure, cardioscope (Lead II), and pulse-oximeter. He was premedicated with inj. ondansetron 4 mg i.v., inj midazolam 2.0 mg i.v. and inj. tramadol 100 mg i.v. the patient was induced with inj. thiopentone sodium 250 mg i.v., relaxed with inj. vecuronium 5 mg i.v. and orotracheal intubation was done with a disposable endotracheal tube of size 8. Patient’s vitals remained stable over this duration (PR: 76 and BP: 110/ 70 mmHg).

The patient was cleaned, draped and surgeon infiltrated the nasal mucosa with 5 ml of adrenaline (1:30000) over 30 s. Immediately after adrenaline infiltration blood pressure became 170/90 mmHg, PR 124/min, irregular. Ventricular ectopics (4-6/min.) were noted. We gave 30 mg of inj. propofol to deepen the plane of anaesthesia. In the mean time we enquired the surgeon about concentration of adrenaline infiltration and any chance of inadvertent intravenous injection. The concentration of injected adrenaline was 1:30000, prepared by trainee nurse, by diluting one ampoule of adrenaline in 30 mL of normal saline. These changes lasted only for 2-3 min and pulse became regular (86/min, good volume) but BP fell to 60/40 mmHg. The patient was taken on 100% oxygen and legs were elevated. The patient was examined for any signs of anaphylaxis. All the vials and ampoules were checked again. Another intravenous line was secured with 18 G cannula and i.v. fluids were infused rapidly. Intermittent three i.v. boluses of ephedrine 6 mg each were given which lead to only a transient increase in BP (80/56 mmHg, PR 86/min, regular, good volume). Inj. hydrocortisone 200 mg and inj. chlorphenaramine malleate 44.5 mg i.v. were given after no response to above management. Dopamine infusion was started at 10 *μ*g/kg/min. Twelve lead ECG was obtained which showed no ischaemia or dysrhythmia. The patient remained hypotensive for 45 min and 3 units of crystalloid and 300 mL of colloid was infused over this duration. Surgery was concluded in the mean time and patient’s BP was 90/64 mmHg at that time. The residual neuromuscular blockade was reversed with inj. neostigmine (2.5 mg) and inj. glycopyrolate. Endotracheal extubation was done after return of protective reflexes. The patient was shifted to intensive care unit (ICU). Dopamine infusion was stopped after few minutes only as BP remained stable (110/70). The serum sample for quantification of serum tryptase level was sent within 2 h to rule out anaphylaxis. The patient was shifted to ward on next day. In the ward, the patient was investigated for any underlying medical problems which may have lead to sudden hypotension. Problems like Addison disease, hypothyroidism and autonomic dysfunctions (e.g. Shy Drager syndrome) were ruled out by endocrinologist and medicine personnel after clinical examination. The report of the serum tryptase level was obtained which was within normal limits (0.4 ng/mL).

## DISCUSSION

Subcutaneous infiltration of an adrenaline-containing solution is time tested practice used to prolong the duration of action of local anaesthetic, decrease surgical bleeding and allow better visualization of the vascular structures during surgery. Head and neck is a highly vascular area and absorption of adrenaline is rapid.

The side effects of diluted adrenaline solutions may be either due to accidental intravenous, inadvertent higher concentrations or altered pharmacokinetics and pharmacodynamics of the drug. Due to high vascularity of the nasal area, even 2.5-10 *μ*g/mL solutions may cause fall in diastolic blood pressure due to activation of β2 receptors in skeletal muscle. Transient hypertension (lasting 1-5 min), and arrhythmias may result due to inadvertent high concentration (10-20 *μ*g/mL), large volume of solutions, rapid infiltration or accidental intravenous infiltration. It may be the probable cause in our case as 5 mL of 1:30000 adrenaline containing normal saline (prepared by trainee nurse) was infiltrated rapidly in the nasal septum and inferior turbinates leading to rapid rise in plasma concentration of adrenaline. These transient changes may be followed by profound hypotension as vasoconstrictive *α* action dissipates at lower levels and β2-mediated skeletal venodilation may exaggerate the fall in cardiac output. It is further exaggerated if patient is already anaesthetized as there will be blunting of sympathetic activity. Therefore, as supported by Li Wei-yan and others lighter plane of anaesthesia may be protective against adrenaline-induced hypotension.[9]

As already mentioned, various studies support that transient hypotension may follow after infiltration of dilute concentration of adrenaline (1:50,000-1:4,00,000). But, to best of our knowledge we have first time encountered such unusual case of prolonged hypotension. The cause for this prolonged effect remains unclear. One probability is that due to vasoconstriction, adrenaline will be continuously absorbed at slower rates (< 4 *μ*g/min) leading to continuous β2 receptor activation. Altered pharmacokinetics may also be a cause for prolonged absorption. The increased β2 receptor sensitivity may also be one other reason for profound hypotension. Another remote possibility may be the occurrence of Bezold –Jarisch Reflex which is a cardioprotective reflex.[10] It is characterized by hypotension, relative bradycardia and coronary vasodilation. Sudden hypertension and tachycardia leading to myocardial insult may have stimulated this reflex in our case. Serum tryptase levels were done in the immediate postoperative period, which were within normal limits. On the basis of history and clinical examination, there were no other findings except hypotension in favour of anaphylaxis.

Above all, circumstantial evidence of symptoms subsequent to subcutaneous adrenaline infiltration goes in support of implication of adrenaline in this episode of unusual prolonged hypotension.

## CONCLUSION

Today, in the era where surgeons are much concerned about minimization of surgical site bleed, anaesthetists must remain cautious while infiltration of adrenaline. Once sudden hypotension occurs in the intraoperative period which is a great challenge to anaesthetist due to array of differential diagnosis, this complication should also be kept in mind.

In nutshell, cardioscopic monitoring should always be considered, whenever cases are done under local anaesthesia containing adrenaline solutions and this complication should always be considered once sudden hypotension develops. We should remain vigilant whenever these techniques are used in conjunction with adrenaline infiltration as precipitous fall in blood pressure may occur.
